# The Immunosuppressive Agent Mizoribine Monophosphate Is an Inhibitor of the Human RNA Capping Enzyme

**DOI:** 10.1371/journal.pone.0054621

**Published:** 2013-01-17

**Authors:** Frédéric Picard-Jean, Isabelle Bougie, Satoshi Shuto, Martin Bisaillon

**Affiliations:** 1 Département de Biochimie, Faculté de Médecine et des Sciences de la Santé, Université de Sherbrooke, Sherbrooke, Québec, Canada; 2 Graduate School of Pharmaceutical Sciences, Hokkaido University, Sapporo, Japan; Kantonal Hospital St. Gallen, Switzerland

## Abstract

Mizoribine monophosphate (MZP) is a specific inhibitor of the cellular inosine-5′-monophosphate dehydrogenase (IMPDH), the enzyme catalyzing the rate-limiting step of *de novo* guanine nucleotide biosynthesis. MZP is a highly potent antagonistic inhibitor of IMPDH that blocks the proliferation of T and B lymphocytes that use the *de novo* pathway of guanine nucleotide synthesis almost exclusively. In the present study, we investigated the ability of MZP to directly inhibit the human RNA capping enzyme (HCE), a protein harboring both RNA 5′-triphosphatase and RNA guanylyltransferase activities. HCE is involved in the synthesis of the cap structure found at the 5′ end of eukaryotic mRNAs, which is critical for the splicing of the cap-proximal intron, the transport of mRNAs from the nucleus to the cytoplasm, and for both the stability and translation of mRNAs. Our biochemical studies provide the first insight that MZP can inhibit the formation of the RNA cap structure catalyzed by HCE. In the presence of MZP, the RNA 5′-triphosphatase activity appears to be relatively unaffected while the RNA guanylyltransferase activity is inhibited, indicating that the RNA guanylyltransferase activity is the main target of MZP inhibition. Kinetic studies reveal that MZP is a non-competitive inhibitor that likely targets an allosteric site on HCE. Mizoribine also impairs mRNA capping in living cells, which could account for the global mechanism of action of this therapeutic agent. Together, our study clearly demonstrates that mizoribine monophosphate inhibits the human RNA guanylyltransferase *in vitro* and impair mRNA capping *in cellulo*.

## Introduction

The synthesis and maturation of eukaryotic mRNAs are crucial events for gene expression. Following mRNA synthesis, eukaryotic mRNAs undergo a series of critical modifications before being exported to the cytoplasm where they are translated into proteins. These processing events include the addition of a cap structure at the 5′ terminus, the splicing out of introns, the editing of specific nucleotides, and the acquisition of a poly(A) tail at the 3′ terminus. The cap structure found at the 5′ end of eukaryotic mRNAs is critical for the splicing of the cap-proximal intron, the transport of mRNAs from the nucleus to the cytoplasm, and for both the stability and translation efficiency of mRNAs [1]. Synthesis of the cap structure occurs co-transcriptionally on nascent mRNAs and involves three enzymatic reactions. First, an RNA 5′-triphosphatase (RTase) hydrolyzes the γ-phosphate at the 5′-end of the nascent pre-mRNA to generate a 5′-diphosphate end. An RNA guanylyltransferase (GTase) then catalyzes a two-step reaction in which it initially utilizes GTP as a substrate to form a covalent enzyme-GMP (EpG) intermediate, with the concomitant release of pyrophosphate (PPi). The GMP moiety is then transferred to the 5′-diphosphate end of the nascent RNA transcript in the second step of the reaction to form the GpppRNA structure. Finally, using S-adenosyl-methionine as a substrate, an RNA (guanine-N7) methyltransferase catalyzes the transfer of a methyl group to the N-7 position of the guanine to produce the characteristic ^m7^GpppRNA cap structure [2]. In humans, a bifunctional RNA capping enzyme catalyzes both the RTase and GTase reactions through distinct domains, while a separate polypeptide mediates the subsequent N-7 methylation [3]. The importance of the cap structure for RNA metabolism is highlighted by genetic analyses in *Saccharomyces cerevisiae* that showed that the triphosphatase, guanylyltransferase and methyltransferase components of the capping apparatus are essential for cell growth [4], [5], [6].

Nascent mRNA capping is a rapid, dynamic, and regulated co-transcriptional process that is subjected to quality control. Transcription initiation is associated with the RNA polymerase II (RNA Pol II) carboxy-terminal domain (CTD) Ser 5 phosphorylation, which recruits the capping apparatus [7]. Nascent mRNAs are rapidly capped (as they are only 20–30 nt long), followed by RNA Pol II CTD Ser 2 phosphorylation, HCE dissociation and mRNA elongation [8]. Messenger RNA capping represents a quality control checkpoint as uncapped RNA are degraded by the Xrn2 5′→3′ exonuclease in order to avoid generation of uncapped mRNA which are not likely to be translated [9], [10], [11]. Uncapped mRNAs are not recognized by the initiation factor eIF4E and are degraded by the 5′→3′ Xrn1 [12], [13]. Given that the RNA Pol II synthesizes 10–30 bases per second, the entire fate of an unsuccessfully capped mRNA can be sealed within few seconds, stressing the importance of rapid and efficient mRNA capping [14].

**Figure 1:**
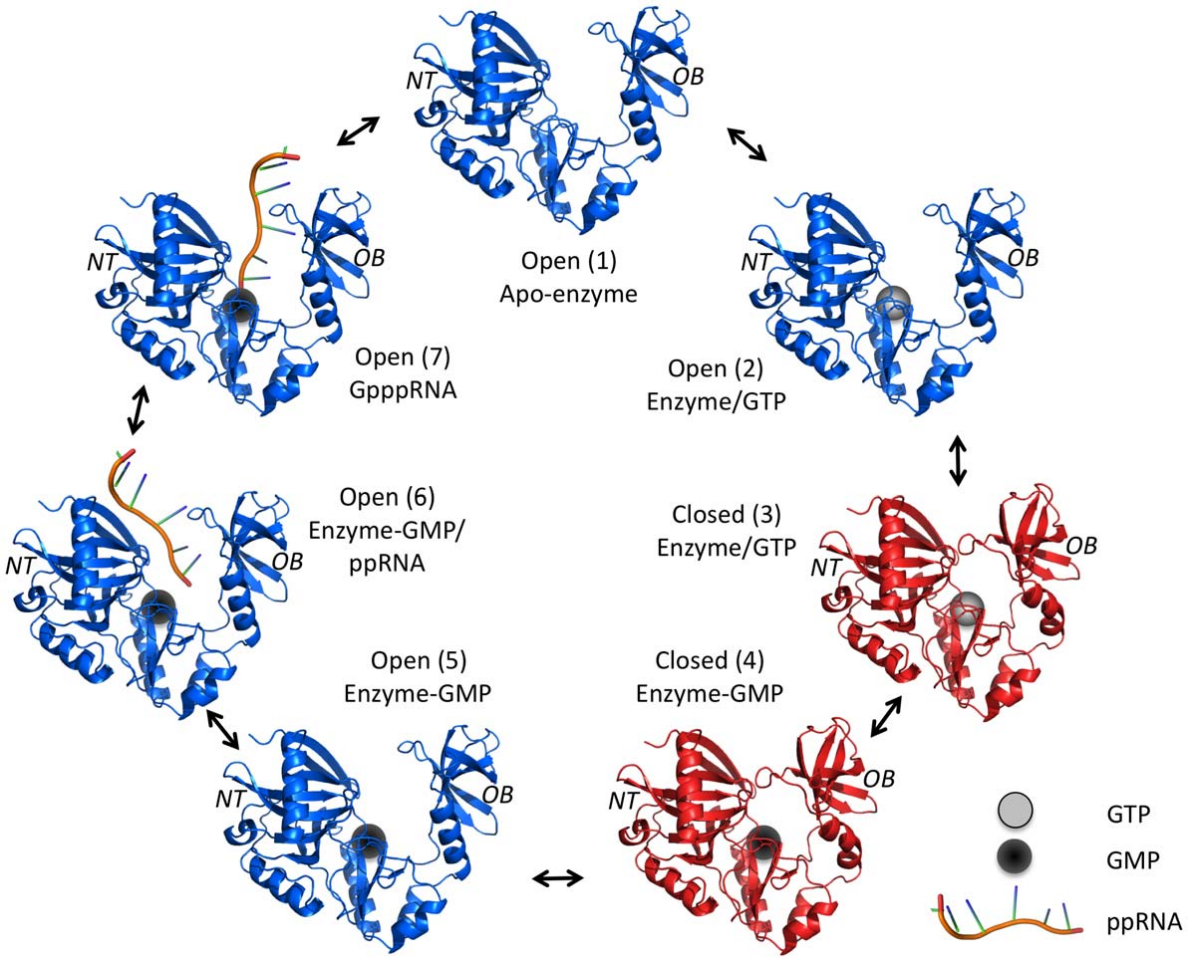
RNA guanylyltransferase mechanistic pathway and associated structures. The phosphoryltransfer catalysis requires conformational changes between the open (blue) and close (red) form of the RNA guanylyltransferase enzyme. The apo-enzyme (structure 1) first binds GTP (grey sphere, structure 2) which promotes the closure of the OB fold domain toward the NT domain (structure 3). In the catalytically active close conformation, the enzyme hydrolyzes the GTP to form the hallmark enzyme-GMP covalent intermediate complex (black sphere, structure 4). The lost of interactions between the bound guanylate and the OB fold domain, upon GTP hydrolysis, destabilizes the close conformation of the enzyme and leads to its reopening (structure 5) concomitant with the release of the pyrophosphate product. This exposes the RNA-binding site of the enzyme (exact location unknown), thereby allowing 5′-diphosphate RNA binding (structure 6) and subsequent GMP moiety transfer onto the acceptor RNA (structure 7). The capped RNA is then released and the apo-enzyme (structure 1) is regenerated allowing reinitiation of the pathway. (PDB: 1CKN).

The rate-limiting activity of the capping apparatus is the two-step ping-pong GTase activity [15], [16]. A general mechanism for phosphoryltransfer involving conformational changes between an open and closed form of the enzyme has been previously solved based on various GTases crystal structures [17], [18]. The first step of the reaction is initiated by the binding of GTP to the open form of the enzyme followed by the closure of the C-terminal oligomer-binding (OB) fold domain and the N-terminal nucleotidyl transferase (NT) domain. This closure is stabilized by interactions between the bound nucleotide and residues from both NT and OB fold domain. Once in the catalytically active close conformation, the GTP substrate is hydrolyzed to produce the enzyme-GMP covalent intermediate. Interactions between the bound guanylate and the OB fold domain are disrupted upon GTP hydrolysis, which leads to the reopening of the enzyme concomitant with the release of pyrophosphate. The open conformation exposes the RNA-binding site, thereby allowing the subsequent transfer of the GMP moiety onto the acceptor RNA. Figure 1 summarizes the mechanistic and structural pathway used by GTases.

Very few inhibitors of the GTase activity have been identified. Recent *in vitro* studies have shown that foscarnet, an antiviral drug that targets the DNA polymerase of human cytomegalovirus, is a potent inhibitor of the GTase reaction [19]. Inhibition of the GTase reaction likely occurs through binding of foscarnet to the active site of the enzyme on account of its analogous nature to pyrophosphate, a product of both the polymerase and the RNA guanylyltransferase reaction. The intracellular triphosphorylated form of ribavirin, a broad-spectrum antiviral nucleoside analogue, can also inhibit the activity of viral GTases. Mechanistic studies have demonstrated that ribavirin triphosphate can actually be used as a substrate by viral GTases [20]. However, RNAs capped with ribavirin are relatively inert to methylation by viral RNA (guanine-N7) methyltransferases, thus resulting in mRNAs that are stable but not efficiently translated into viral proteins [21]. Ribavirin is a pleiotropic agent that can also inhibit the cellular inosine-5′-monophosphate dehydrogenase (IMPDH), which is critical in the metabolism of nucleic acid precursors [22]. The enzyme catalyzes the conversion of inosine monophosphate (IMP) to xanthosine monophosphate (XMP) with the concomitant reduction of NAD via a covalent intermediate (E-XMP). This conversion has been shown to be the rate-limiting step in *de novo* guanine nucleotide biosynthesis [23]. This reduction in intracellular concentrations of guanosine is a key factor which contributes to the decrease in viral replication [22].

**Figure 2:**
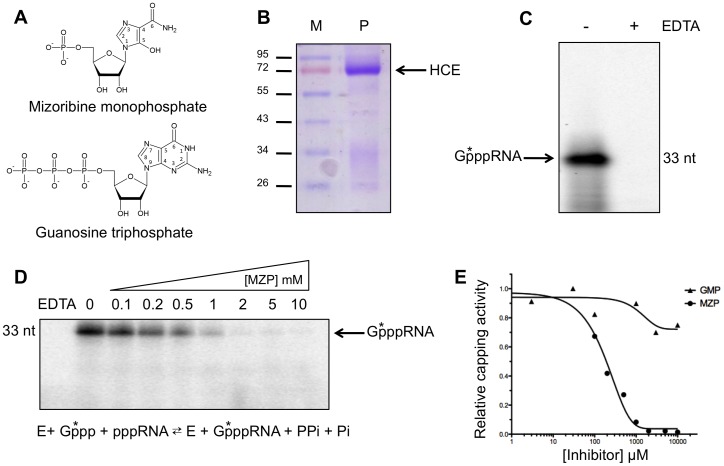
Inhibition of capping by MZP. (A) Structure of mizoribine 5-monophosphate (MZP) and guanosine-5′-triphosphate (GTP). (B) An aliquot of the purified HCE protein (P) was analyzed by electrophoresis through a 12.5% polyacrylamide gel containing 0.1% SDS and visualized by staining with Coomassie blue dye. The position and size (in kDa) of the molecular weight marker (M) are indicated on the left. (C) RNA capping assay by the sequential RTase and GTase activity of HCE. A 5′-triphosphate RNA and [α-^32^P]GTP were incubated in presence of HCE in a buffer containing 5 mM MgCl_2_. As a control, EDTA was added to a final concentration of 50 mM to prevent RNA capping. Radiolabeled RNA products were analyzed by SDS-PAGE, an autoradiogram of the gel is shown. (D and E) Capping assays were performed in the presence of increasing concentration of MZP or GMP and the capped RNA products were analyzed by SDS-PAGE and quantified by autoradiography.

Mizoribine monophosphate (MZP) is another compound which has been shown to specifically inhibit the cellular IMPDH [24]. MZP (Fig. 2A) is the active metabolite of the immunosuppressive pro-drug mizoribine (Bredinin), a nucleoside analog of the imidazole class that was originally isolated from *Eupenicillium brefeldianum* [25], [26], [27]. Crystallographic studies have demonstrated that the binding of MZP to IMPDH results in a transition state analogue complex reminiscent of the E-XMP intermediate formed during the conversion of IMP to XMP [25]. MZP is therefore a highly potent antagonistic inhibitor of IMPDH that blocks the proliferation of T and B lymphocytes that use the *de novo* pathway of guanine nucleotide synthesis almost exclusively. Guanosine nucleotide depletion impairs G protein coupled receptor transduction and cyclic GMP signaling, ultimately resulting in the interruption of the S phase of the cell cycle through specific inhibition of IMPDH [28]. The use of mizoribine has been approved in Japan for induction and maintenance of immunosuppressive therapy after renal transplantation [29].

Since ribavirin triphosphate and MZP share functional similarities, we investigated the attractive possibility that MZP could also inhibit the GTase activity of the human RNA capping enzyme (HCE). In the present study, we demonstrate that MZP can inhibit the formation of the RNA cap structure catalyzed by HCE. The biological implications of this inhibition are discussed.

## Materials and Methods

### Expression and Purification of Recombinant HCE Protein

An expression plasmid containing the full-length HCE protein (597 amino acids) was generated by inserting the corresponding cDNA between the *Nhe*I and *XhoI* cloning sites of the pET28a plasmid (Novagen). In this context, the HCE protein is fused in frame with an N-terminal 6-His tag, and expression of the protein is driven by a T7 RNA polymerase promoter. Upon transformation of the pet-HCE plasmid into *Escherichia coli* BL21(DE3), cultures were grown at 37°C in Luria-Bertani medium containing 30 µg/mL kanamycine until the *A*_600_ reached 0.5. Induction was initiated with 400 µM isopropyl β-d-thiogalactopyranoside (IPTG) and 2% ethanol, protein expression was allowed for 20 h at 18°C. All subsequent procedures were performed at 4°C. The bacteria were harvested by centrifugation at 5000 rpm and resuspended in 50 mL of lysis buffer [50 mM Tris-HCl (pH 7.5), 150 mM NaCl, and 10% sucrose]. 50 µg/mL lysozyme and 0.1% Triton were added prior to sonication. After removal of insoluble material by centrifugation at 13,000 rpm, the soluble extract was applied to a nickel−nitrilotriacetic acid−agarose (Qiagen) column. The protein was eluted stepwise with elution buffer [50 mM Tris-HCl (pH 8.0), 100 mM NaCl, and 10% glycerol] containing 50, 100, 200, 500, and 1000 mM imidazole. The polypeptide composition of the fractions was monitored by SDS-PAGE. The recombinant HCE protein was recovered in the 200 mM imidazole eluate. This fraction was dialyzed against buffer C [50 mM Tris-HCl (pH 8.0), 50 mM NaCl, 2 mM dithiothreitol (DTT), and 10% glycerol] that was supplemented with 5 mM potassium pyrophosphate to ensure a homogeneous non-guanylylated enzyme. The protein concentration was determined by the Bio-Rad dye binding method and stored at −80°C. The amino acids 1–219 corresponding to HCE RTase domain (HCE-T_1–219_) and 229–597 corresponding to HCE GTase domain (HCE-G_229–597_) were also expressed separately using the same procedure.

### Synthesis of the RNA Substrates

An RNA substrate of 32 nucleotides (5′-GGGCACACACAGTCGACCACACAAAACCACCC-3′) was synthesized with the MAXIscript kit (Ambion) using T7 RNA polymerase. The 5′-triphosphate RNA substrate was purified on a denaturing 8% polyacrylamide gel and visualized by ultraviolet shadowing. The corresponding band was excised and then eluted from the gel by an overnight incubation in 0.1% SDS and 0.5 M ammonium acetate. The RNA was then precipitated with ethanol and quantitated by spectrophotometry. Alternatively, radiolabeled RNA substrates were also synthesized by adding radiolabeled nucleotides to the transcription reaction.

A 5′-diphosphate RNA substrate was also synthesized by subjecting the 5′-triphosphate RNA to hydrolysis by the *Saccharomyces cerevisiae* RTase (Cet1). The reaction was carried out in a buffer containing 50 mM Tris-HCl, pH 7.5, 1 mM MgCl_2_, 5 mM DTT, 1 µM of Cet1, and 5 nmoles of the 5′-triphosphate RNA for 30 min at 30°C. This 5′-diphosphate RNA was then purified on an 8% polyacrylamide gel, excised, and precipitated. The RNA substrate was quantified by spectrophotometry and stored at −20°C.

### Capping of a 5′-triphosphate RNA

Reaction mixtures containing 5 µM [α-^32^P]GTP, 5 µM of 5′-triphosphate RNA, 0.1 µM HCE, 50 mM Tris-HCl, pH 7.5, 5 mM MgCl_2_, 500 µM DTT, 0.5 ng/µl pyrophosphatase (Roche), and 1 U of RNAse Out (Invitrogen) were incubated for 15 min at 37°C. Reactions were stopped by the addition of 20 µl of phenol-chloroform. 10 µl of loading dye (97% formamide) was added to the aqueous phase and submitted to electrophoresis on a denaturing 10% polyacrylamide gel. Formation of the ^32^P-radiolabeled GpppRNA was quantified with a PhosphorImager.

### RNA Triphosphatase Assay

Reaction mixtures containing 50 mM Tris-HCl, pH 7.5, 5 mM MgCl_2_, 500 µM DTT, 3 µM of γ-^32^P-radiolabeled 5′-triphosphate RNA substrate (32 nt), 0.1 µM HCE, and various concentration of MZP (as indicated) were incubated for 10 min at 37°C. The reactions were stopped by the addition of 2 µl of 5 M formic acid. Aliquots of each sample were analyzed on polyethyleneimine-cellulose thin-layer chromatography (TLC) plate, and developed with 0.5 M LiCl and 1 M formic acid. The release of ^32^Pi was quantified by scanning the TLC plates with a PhosphorImager.

### RNA Guanylyltransferase Assay

GTase assays were conducted as described in the “capping of a 5′-triphosphate RNA” section except that a 5′-diphosphate RNA was use as a substrate. Alternatively HCE was substituted by either 0.08 µM D1R (Vaccinia virus), 0.4 µM HCE-G_229–597_, 1.2 µM A103R (*Chlorella* virus) or 0,5 µM Ceg1 (*S. cerevisiae*).

### Enzyme-GMP Complex Formation

The enzyme-GMP complex formation assays were carried out for 3 min at 37°C in a buffer containing 1 µM [α-^32^P]GTP, 4 µM HCE, 50 mM Tris-HCl, pH 7.5, 5 mM MgCl_2_, 500 µM DTT, 0.5 ng/µl pyrophosphatase (Roche), and various concentration of MZP (as indicated). The reactions were stopped by the addition of 5 µl of loading dye (50 mM EDTA, 2% β-mercaptoethanol, 1% SDS) and analyzed on a 12% polyacrylamide gel containing 0.1% SDS. The formation of the radiolabeled HCE-[α-^32^P]GMP covalent complex was quantified with a PhosphorImager. Similar experiments were conducted using only the GTase domain of HCE (HCE-G_229–597_). Alternatively, equimolar concentrations of MgCl_2_ to MZP were added to the reaction in addition to the base 5 mM MgCl_2_ from the buffer (MZP✯Mg^2+^).

### GMP Transfer on a 5′-diphosphate RNA

Formation of the enzyme-GMP complex was initially performed for 15 min at 37°C in a buffer containing 1.5 µM [α-^32^P]GTP, 1 µM HCE, 70 mM Tris-HCl, pH 7.5, 7.5 mM MgCl_2_, 70 µM DTT, 0.7 ng/µl pyrophosphatase (Roche), and 1 U of RNAse Out (Invitrogen). The transfer reaction was then initiated by adding a 5′-diphosphate RNA (32 nt) to a final concentration of 0.5 µM with various concentration of MZP (as indicated). After 30 sec at 37°C, the transfer reaction was stopped by the addition of 20 µl of phenol-chloroform. 10 µl of loading dye (97% formamide) was added to the aqueous phase and submitted to electrophoresis on a denaturing 10% polyacrylamide gel. The formation of the ^32^P-radiolabeled GpppRNA was quantified with a PhosphorImager.

### MZP Transfer onto an Acceptor RNA

Reaction mixtures containing 50 mM Tris-HCl, pH 7.5, 5 mM MgCl_2_, 500 µM DTT, 0.7 ng/µl pyrophosphatase (Roche), and 1 U of RNAse Out (Invitrogen), 1 µM of 5′-diphosphate RNA substrate (32 nt) harboring a α-^32^P-radiolabeled 5′-phosphate, 0.2 µM HCE were incubated in absence or in presence of 2 mM GTP or MZP for 2 h at 37°C. The reaction pH was adjusted to 5.2 with 50 mM NaOAc followed by the addition of 5 µg of Nuclease P1 and incubation at 37°C for 60 min. The digestion were further adjusted with 50 mM Tris-HCl to pH 8.0, and digested with 1 U of alkalie phosphatase (Roche) for 30 min at 37°C. The reaction products were analyzed by thin-layer chromatography (TLC) on a polyethyleneimine-cellulose plate developed with 0.4 M ammonium sulfate.

### Inhibition of mRNA Cap Formation in Cells

HEK 293T cells were cultured in Dulbecco’s minimum essential medium (DMEM) supplemented with 10% fetal calf serum. Cell were transfected according to the manufacturer instruction using GeneCellin reagent (BioCellChallenge, Toulon, France). On day 1, 70% confluent 10 cm petri dishes where transfected with either pCDNA3.1+ (Invitrogen) (Control Vector), pCDNA3.1+ construct expressing either the full-length HCE wild-type protein (557 amino acids) fused to an HA tag (HCE-WT-HA), the GTase defective HCE K294A mutant fused to an HA tag (HCE-K294A-HA), or the green fluorescent protein (GFP). Eighteen hours later, 5×10^4^ transfected cells were seeded per well on 24-well tissues cultures plates. At 24 h mizoribine (Sigma) was added to a final concentration of 0, 40, or 120 µM. At 66 h cells were submitted to a second transfection using the firefly luciferase encoding vector PGL3 (Promega) and were harvested at 96 h to be submitted to luciferase assay (Promega) according to manufacturer instructions. Luciferase activities were normalized to untreated cells. Western blot were performed using 10 µg of total protein extracted at 96 h. The protein were separated on a 12% SDS-PAGE and transferred onto a polyvinylidene fluoride membrane. The membrane was blocked and incubated overnight with either 1∶1000 anti-HA (HA-probe (F-7), sc-7392, Santa Cruz Biotechnology), 1∶1000 anti-GFP (G1546, Sigma-Aldrich), or 1∶10000 anti-Actin (β-Actin Antibody #4967, Cell Signaling) primary monoclonal antibody followed by 1 h incubation with 1∶5000 HRP-anti-mouse (GE Healtcare) secondary antibody. Revelation was done using ECL detection (PerkinElmer) and exposure to x-ray film (Denville Scientific).

## Results

### RNA Capping Activity

Metazoans initiate mRNA capping with a bifunctional enzyme that harbors the RTase and GTase activity on the same peptide. In order to evaluate the inhibitory potency of MZP on the human capping apparatus, a hexa-histidine tagged full-length bifunctional HCE protein (amino acid 1–597) was expressed in *E. coli* and purified by nickel-agarose chromatography. To ensure the purification of the apo-enzyme, HCE was further dialyzed against potassium pyrophosphate and magnesium to remove any residual HCE-GMP complex. The 69-kDa HCE protein was the predominant polypeptide in the purified fraction (Fig. 2B). In the presence of a 32-nt 5′-triphosphate RNA, [α-^32^P]GTP and magnesium cofactor, the full-length HCE was also shown to sequentially hydrolyze the γ-phosphate from the RNA substrate and transfer the GMP moiety from the GTP substrate onto the newly 5′-diphosphorylated RNA to form the GpppRNA cap structure (Fig. 2C).

### Capping Inhibition by MZP

In order to investigate if MZP could inhibit the RNA capping reaction, we initially addressed its impact on the complete RNA capping reaction (RTase+GTase). A cold 5′-triphosphate RNA was incubated in the presence of HCE, [α-^32^P]GTP, magnesium and increasing concentrations of MZP. As shown in figure 2D, the addition of increasing concentrations of MZP to the reaction efficiently prevented the formation of the radiolabeled capped RNA. MZP inhibited the reaction with an IC_50_ of 150 µM (Fig. 2E). To ensure that the inhibition by MZP was specific, the reaction was performed in the presence of increasing concentrations of GMP which is chemically related to MZP. The RNA capping reaction was not significantly altered by the presence of increasing concentrations of GMP; concentrations as high as 10 mM did not reduce the RNA capping by more than 25% (Fig. 2E).

### MZP does not Inhibit the RTase Reaction

**Figure 3:**
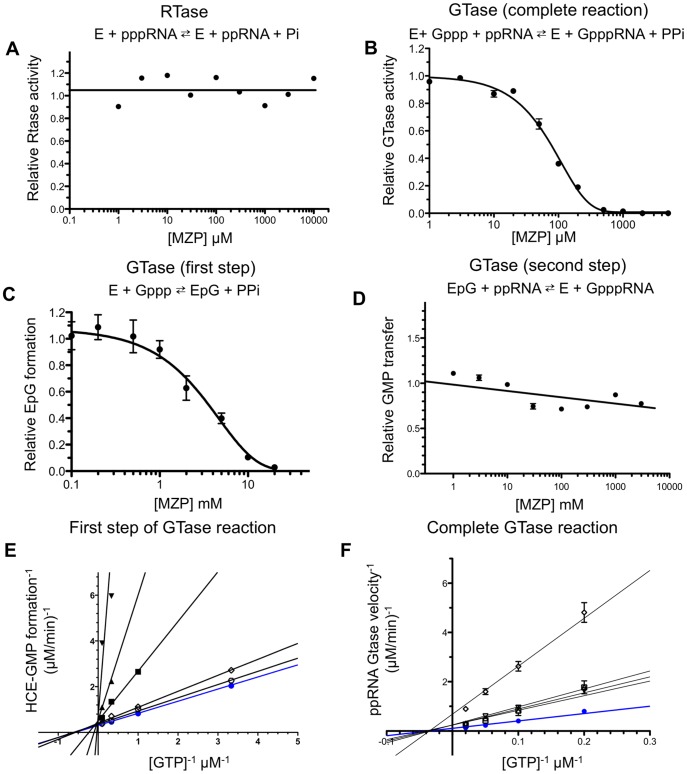
MZP mechanism of inhibition. (A) MZP does not inhibits the RTase activity. An RNA substrate radiolabeled at its 5′-terminal γ-phosphate was incubated with HCE and increasing concentration of MZP. ^32^Pi product was separated from RNA substrate by polyethyleneimine-cellulose TLC plate and quantified by autoradiography. (B) The GTase activity is inhibited by MZP. A 5′-diphosphate RNA was incubated with [α-^32^P]GTP, HCE and increasing concentration of MZP. Radiolabeled RNA products were analyzed on denaturing polyacrylamide gel and quantified by autoradiography. (C) The formation of HCE-GMP complex, the first step of GTase activity, is inhibited to a lesser extent then the complete GTase reaction by MZP. HCE was incubated with [α-^32^P]GTP and increasing concentration of MZP. Radiolabeled EpG complex were analyzed by SDS-PAGE and quantified by autoradiography. (D) MZP does not significantly inhibit the transfer of a GMP on an acceptor RNA; the second step of GTase activity. HCE was preincubated with [α-^32^P]GTP to allow EpG formation, a 5′-diphosphate RNA and increasing concentration of MZP were next added to the reaction. Formation of radiolabeled RNA products was analyzed on denaturing polyacrylamide gel and quantified by autoradiography. (E) Lineweaver-Burk representation of the enzyme-GMP covalent complex formation (GTase first step) as a function of the substrate concentration in presence of various MZP concentration. The maximal velocity of EpG formation is not affected by various MZP concentration (curves crossing on the Y axis). (F) Lineweaver-Burk representation of the complete GTase reaction velocity as a function of the substrate concentration in presence of various MZP concentration. HCE K_m_ for GTP is independent from the MZP concentration (curves crossing on the X axis) for the complete reaction. MZP concentration : ✯ 0 µM, □ 50 µM, ▵ 200 µM, ▿ 500 µM, ✵ 1 mM, ★ 2 mM, ▪ 5 mM, ▴ 10 mM, ▾ 20 mM.

We next investigated the susceptibility of the RTase domain of HCE to MZP inhibition. To specifically monitor the effect of MZP on the RTase activity, a γ-^32^P-radiolabeled 5′-triphosphate RNA was incubated with HCE and magnesium. RTase activity was monitored through the analysis of the ^32^Pi product released from the radiolabeled RNA by TLC and autoradiography. Our data indicate that increasing concentrations of MZP does not affect the RTase activity up to 10 mM (Fig. 3A). It should be noted that similar results were obtained when the assay was performed with only the RTase domain of HCE (HCE-T_1–219_) (data not shown).

### MZP Inhibits the GTase Reaction

**Figure 4:**
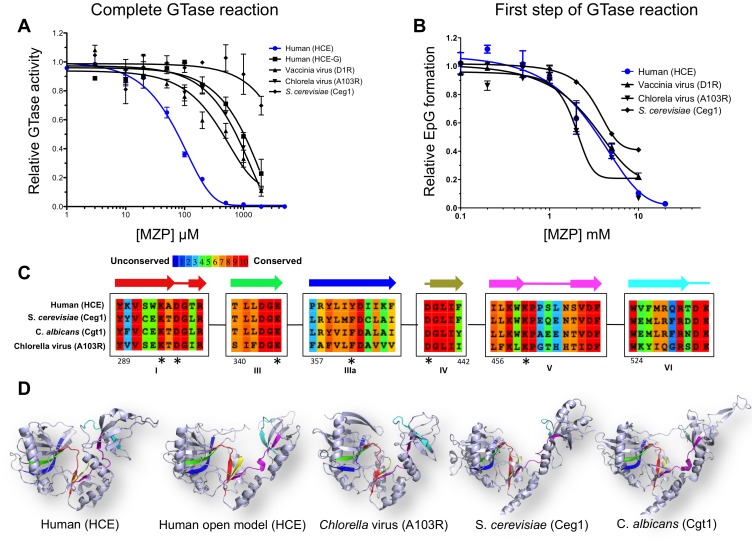
Specificity of MZP inhibition toward RNA guanylyltransferase. (A) Complete GTase reaction. 5′-diphosphate RNA was capped with HCE, HCE-G_229–597_, D1R, A103R or Ceg1 GTases in presence of increasing concentration of MZP. (B) Formation of EpG complex. The GTases from human, vaccinia virus, *Chlorella* virus or *S. cerevisiae* were allowed to form enzyme-GMP covalent complex in presence of increasing concentration of MZP. (C) Amino acids alignment of the human (HCE), *S. cerevisiae* (Ceg1), *C. albicans* and *Chlorella* virus (A103R) GTases highlighting the sequence conservation. Representation of the six conserved GTase signature motifs (I, III, IIIa, IV, V, VI) and their associated secondary structures (above). Numbering is based on the HCE amino acids. Amino acids predicted to coordinate MZP low affinity binding site are highlighted by an asterisk. (D) GTases harbor a similar three-dimensional architecture. Ribbon diagram of HCE GTase domain in close conformation (PDB: 3S24), in open conformation (homology model base on PDB: 1P16), *Chlorella* virus GTase (PDB: 1CKN), *S. cerevisiae* GTase (PDB: 3KYH), *C. albicans* GTase (PDB: 1P16). Active site color code is representative of the GTase signature motif as displayed in panel C.

The GTase reaction is a two-step ping-pong reaction, which involves the formation of a covalent EpG intermediate upon GTP hydrolysis, followed by the transfer of this GMP moiety onto a 5′-diphosphate RNA. We initially investigated the inhibitory potential of MZP on the complete two-step GTase reaction in steady-state condition (multiple turnover). A 5′-diphosphate RNA, [α-^32^P]GTP, magnesium, pyrophosphatase and various concentration of MZP were preincubated together, and the reaction was initiated by the addition of HCE. A concentration of 80 µM MZP resulted in a 50% decrease in the formation of the radiolabeled capped RNA (Fig. 3B). Interestingly, when the same experiment was conducted with the isolated GTase domain of HCE (HCE-G_229–597_) the activity was still inhibited by MZP, albeit with a 10-fold decrease in the potency (Fig. 3B and 4A). In order to address if it was the presence (or the activity) of the RTase domain that could influence the susceptibility of the HCE GTase domain to MZP inhibition, we generated a point mutation of the catalytic Cysteine 126 of the RTase domain from the classical phosphatase core motif (I/V)HCxxGxxR(S/T)G [30]. Our results show that this RTase-defective full-length HCE mutant (HCE-C126S) displayed a similar inhibition profile than the wild-type enzyme (Fig. S1). Consequently, we conclude that the GTase reaction represents the catalytic step which displays the highest susceptibility to MZP inhibition during the synthesis of the RNA cap structure.

### Mechanism of the GTase Inhibition

We next set out to identify which step (if any) of the GTase reaction is more susceptible to MZP inhibition. In order to investigate the inhibitory potency of MZP on HCE-GMP complex formation (first step), HCE was incubated with [α-^32^P]GTP, magnesium and increasing concentrations MZP. Since this GTase reaction is known to be highly inhibited by its pyrophosphate product, pyrophosphatase was also added to the reaction in order to drive the reaction forward. The formation of the EpG covalent complex in single turnover condition was analyzed by SDS-PAGE and quantified by phosphorimaging. As seen in figure 3C, increasing concentrations of MZP only prevented formation of the EpG complex at elevated concentrations of MZP; a concentration of 3 mM MZP resulted in a 50% inhibition of the HCE-GMP complex formation. Despite the low millimolar MZP concentration required to prevent the EpG formation, this inhibition is not due to magnesium cofactor sequestration since the addition of an equimolar concentrations of MgCl_2_ to MZP did not influence this effect (data not shown). Nevertheless, MZP inhibition of the EpG complex formation is weak and could not be solely responsible for the global inhibitory potency of MZP.

We next investigated if the second step of the GTase reaction could be impaired by MZP. EpG complex formation was allowed to initially form upon incubation of HCE with excess [α-^32^P]GTP, magnesium and pyrophosphatase. Next, various concentrations of MZP and a cold 5′-diphosphate RNA were added to initiate the transfer of the [α-^32^P]GMP moiety onto the RNA in single turnover condition. As seen in figure 3D, our data indicate that MZP only has a minor impact on the second GTase step. We conclude that the inhibition of this step does not contribute significantly to the general inhibition effect caused by MZP.

Quantitative analysis of the MZP inhibition for the complete GTase reaction, or its first step alone, were conducted. The formation of GpppRNA structure or the EpG covalent complex was monitored as a function of GTP concentration in the presence of increasing concentrations of MZP. The MZP concentration had no impact on the maximal velocity of the EpG formation (GTase first step), which suggested a competitive mechanism of inhibition (Fig. 3E). Interestingly, for the complete GTase reaction, the K_m_ appears to be independent from the inhibitor concentration, which would suggest a non-competitive mechanism of inhibition (Fig. 3F). It should be noted that HCE was not able to use the MZP as a substrate and transfer it onto a radiolabeled acceptor RNA to form an atypical cap structure (Fig. S2). These results highlight 2 potentially different mechanisms of action of MZP. The weak inhibition of the EpG formation appears to be competitive while the global inhibition seems to be non-competitive. The later raises the possibility that MZP could primarily bind to an allosteric binding site.

### MZP Demonstrate Specificity for HCE Over Other GTases

In order to investigate if MZP specifically inhibits HCE, and gain additional insight on the degree of conservation of the main MZP binding site, we conducted a wide array of GTase inhibition studies. The mammalian vaccinia virus GTase (D1R), the *Saccharomyces cerevisiae* GTase (Ceg1) and the GTase (A103R) from *Chlorella* virus (PBCV-1) were submitted to MZP inhibition for both the complete GTase reaction and the EpG formation (GTase first step). As compared to the HCE (IC_50_ = 80 µM), the other GTases showed reduced susceptibility to MZP inhibition for the complete GTase reaction. This difference ranges from 5-to 25-fold for the GTase from the mammalian vaccinia virus (IC_50_ = 500 µM), the *Chlorella* virus (IC_50_ = 750 µM), and the yeast *S. cerevisiae* (IC_50_>2 mM) (Fig. 4A). MZP also inhibited the EpG formation by all GTase assessed, albeit with smaller variations (Fig. 4B). Both the human and vaccinia virus GTase (HCE and D1R) had IC_50_ values of 3 mM, the *Chlorella* virus GTase (A103R) presented an IC_50_ of 2 mM, while the yeast GTase (Ceg1) seemed slightly less affected by MZP. Unlike the complete GTase reaction, the low potency inhibition of the EpG formation by MZP is fairly similar for all GTases assessed. These results support the hypothesis that the inhibition of the HCE-GMP complex by MZP could only account for a minor contribution to the complete GTase activity inhibition. This raises the possibility that a conformational change hindrance could be the mechanism of action by which MZP inhibits HCE. This hypothesis will be further investigated in the discussion.

### *In cellulo* Capping Inhibition

**Figure 5:**
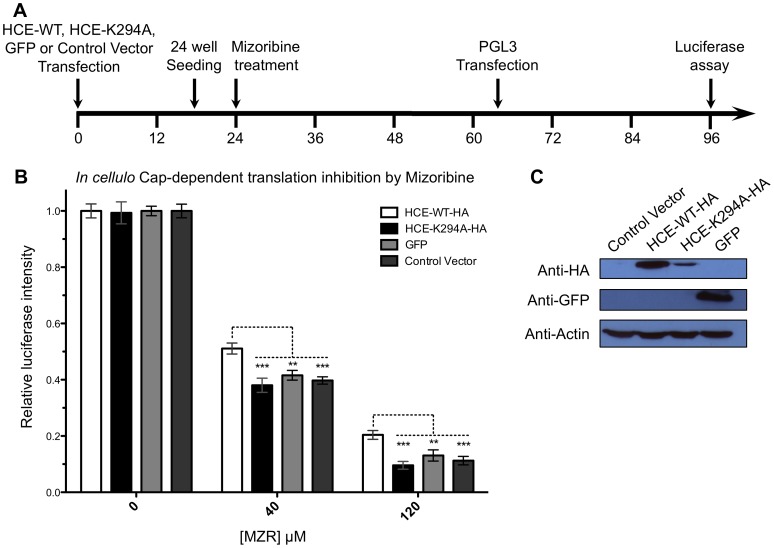
*In cellulo* mizoribine capping inhibition is rescued by HCE over-expression. (A) Timeline of the experiments. (B) Cells over-expressing HCE-WT-HA, HCE-K294A-HA, GFP, or control cells were pre-treated with mizoribine for 42 h and transfected with the luciferase encoding vector PGL3. The RNA pol II transcribe reporter gene was allowed to express for 30 h prior to luciferase assay. Luciferase intensity, as a mesure of efficient capping of the reporter protein mRNA, was normalized for untreated cells and plotted against mizoribine concentration. For both mizoribine concentrations tested, the luciferase activity was significantly higher in cells over-expressing HCE-WT-HA as compared to control cells (cells over-expressing the GTase defective HCE-K294A-HA mutant, over-expressing GFP, or transfected with the control Vector). Shown is the mean of 18 experiments for each condition. (C) Western blots using anti-HA, anti-GFP and anti-actin antibody were performed on protein extracts from each cell line. (**P<0.01, ***P<0.001).

We next set out to investigate if the *in vitro* inhibitory potency of MZP on HCE could be translated into the inhibition of the capping apparatus in living cells. Monitoring the capping efficiency in mammalian cells is a great challenge. RNA quality control systems ensure that unsuccessfully capped mRNAs are rapidly degraded. As a net result, capping inhibition would not lead to uncapped mRNA accumulation, but rather to a global decrease of mature mRNAs. This potentially allows to indirectly evaluate the ability of MZP to inhibit RNA capping by monitoring the transcription and translation of a reporter gene in a controlled environment. On average, an mRNA is transcribed every 30 min, has a half-life of 9 hours, and is translated 40 times per hour to yield an average of 500–1000 proteins over its life span [31]. This gives nearly 3 orders of magnitude of amplification over every capping event and provides a very sensitive assay. As an attempt to rescue the capping inhibition induced by MZP, we chose four identical human embryonic kidney (HEK 293T) cell lines, diverging only by the over-expression (or not) of HCE-WT-HA, HCE-K294A-HA (GTase defective mutant) or GFP protein (Fig. 5C). Unlike HCE-WT-HA, HCE-K294A-HA might be slightly toxic (negative dominant effect), which would explain its lower over-expression, nevertheless, HCE-K294A-HA can be over-expressed in mamalien cells and is easily detectable. Both the activity and the stability of the reporter gene (firefly luciferase) are not influenced by high concentration of HCE (Fig. S3). The mizoribine prodrug was added to a final concentration of 0, 40 or 120 µM to the four cell lines. The reporter gene expression was initiated upon transfection of the PGL3 vector (bearing the firefly luciferase reporter gene under the control of an RNA Pol II promoter). Luminescence quantification of the reporter level was performed after 30 h of reporter protein expression (Fig. 5A). Our results indicate that cells treated with 40 µM or 120 µM mizoribine show a global reduction in protein expression, likely due to the partial GTP depletion induced by IMPDH inhibition. Interestingly, the translation of the reporter gene was partially rescued only in cells over-expressing HCE-WT-HA when compared the HCE-K294A-HA, GFP, and control cells. In the presence of 40 µM and 120 µM of mizoribine, the cells over-expressing HCE-WT-HA maintained a significantly higher reporter gene translation rate compared to all the other cell lines that did not harbor a functional capping apparatus (Fig. 5B). Although this experiment does not allow for precise quantification of the capping inhibition, it demonstrates that the over-expression of the active capping apparatus in human cells was partially able to rescue the mizoribine-induced phenotype on a reporter gene that is dependent on the cap structure for its proper transcription and translation.

## Discussion

Our study provides the first biochemical evidences that mizoribine monophosphate can directly inhibit the human capping enzyme. The ability of MZP to inhibit a purified RNA capping enzyme has not been previously documented and has implications on our understanding of the catalytic mechanism of RNA capping enzyme. Our results indicate that the overall GTase reaction is inhibited by MZP. HCE is a bifunctional protein harboring both RTase and GTase activity. In the presence of MZP, the RTase activity appears to be relatively unaffected while the GTase activity is inhibited, indicating that the GTase activity is the main target of MZP inhibition.

The GTase catalysis is a two-step reaction. Interestingly, the inhibition of the complete GTase reaction by MZP could not be explained completely by the inhibition of its individual steps. While the GMP transfer onto an acceptor RNA (second step) does not contribute significantly to the general inhibition effect caused by MZP, the EpG formation (first step) is inhibited by MZP with an IC_50_ 25-times higher than the complete GTase reaction. This weak inhibition, led us to hypothesize that two distinct MZP binding sites could be present on HCE. The main binding site responsible for the drug inhibitory potency is speculated to be allosteric and will be further discussed. The second would be the active site and is believed to bind MZP with a lower affinity, likely due to the drug chemical similarity with GMP. The weak binding of MZP to the active site would be coherent with EpG complex formation being competitively inhibited by MZP. *In silico* docking of MZP on the highly conserved active site of an open conformation homology model of HCE GTase domain provides additional information on the mechanism of MZP binding to this site (Fig. S4 and Table S1). MZP appears to be coordinated by the most conserved amino acids, which would thereby explain the low inhibition specificity toward various GTase (Fig. 4C and D). Nevertheless, the mechanism of inhibition of the enzyme-GMP complex, which is mediated by the weak binding of MZP to the active site, is fundamentally different and weaker than the mechanism of the complete GTase inhibition. A complete GTase catalytic round does not only imply the formation of the EpG intermediate complex and the transfer of the GMP moiety to an acceptor RNA, but also involves complex conformational changes where the OB fold domain leans toward or away from the NT domain. MZP could bind HCE and block this conformational change, possibly through stabilization of the closed conformation, thereby preventing the reopening of the protein upon GTP hydrolysis. This hypothesis would likely imply an allosteric MZP binding site. Interestingly, the kinetics studies, although performed in steady-state condition, point toward a non-competitive mechanism of inhibition, which would indicate that MZP binds elsewhere than the active site. This is also coherent with the inability of MZP to be used as a substrate and transferred onto an acceptor RNA. Despite the very high degree of conservation among this specific family of nucleotidyltransferases, MZP harbors a 5-to 25-fold gain in specificity for HCE when compared to other GTases. This result additionally supports the presence of an allosteric MZP binding pocket. Oddly enough, the lone GTase domain of HCE (HCE-G_229–597_) is less susceptible by 10-fold to MZP inhibition than the full-length HCE. This evidence, not only supports the presence of an allosteric site, but can also provide additional information about its localization on HCE. It is tempting to speculate that MZP could bind near the N-terminal of the GTase domain (close to the RTase-GTase interdomain) or on a region of interaction between both the RTase and GTase domains since the abolition of the RTase domain reduces HCE MZP susceptibility. In order to gain additional details on the MZP main binding site, molecular docking could be used. Unfortunately, the structure of both the RTase and the GTase domain are separately available, but the structure of the full-length protein is still not available. Nevertheless, we ran a molecular docking experiment of MZP on the GTase domain from HCE. The lack of RTase domain, which is predicted to participate in MZP binding, and the moderate flexibility of the N-terminal domain, which introduces a structural incertitude, does not allow for definitive conclusions to be reached but it is interesting to note that, in preliminary experiments, MZP favorably docks on the N-terminal region of the HCE GTase (near the RTase-GTase inter-domain). Together, our results reveal that MZP inhibits the HCE GTase activity with a 5-to 25-fold specificity in comparison to other GTases. Although more work is yet required to confirm our hypothesis, these results raise the possibility that the GTase inhibition could be mediated by a conformational change hindrance upon binding of MZP to an allosteric binding site that is speculated to reside near the RTase-GTase inter-domain. Nevertheless, mizoribine is one of the first compounds to demonstrate a certain degree of specificity toward a single GTase, despite the high degree of conservation of this crucial family of enzyme.

MZP displays a higher *in vitro* inhibition potency for the GTase reaction (IC_50_ = 80 µM) in comparison to the complete RNA capping reaction (RTase+GTase) (IC_50_ = 150 µM). This may simply be due to our experimental conditions (5 mM Mg^2+^ and low nucleotide triphosphate concentration) where the RTase activity of HCE, which is partially inhibited by free Mg^2+^, becomes the rate-limiting step [30]. However, *in cellulo* the RTase harbors a higher turnover rate than the GTase, which catalyzes the limiting step in RNA capping [15]. In a cellular context we expect the efficiency of MZP to be dictated solely by its interaction with the GTase.

Historically, very few GTase inhibitors have been developed, neither as scientific tools nor as therapeutic agents. More recently however, novel GTase inhibitors have been discovered [32]. They include the allosteric inhibitor mycophenolic acid, the pyrophosphate analog foscarnet which acts as a product inhibitor, and ribavirin triphosphate, a GTP analog that is transferred to acceptor RNAs by GTase, leading to stable but inefficiently translated pseudo-capped RNA. The current study identifies MZP as a novel allosteric GTase inhibitor, which is speculated to block a crucial conformational change. The GTase activity being the rate-limiting step of the essential capping apparatus, all these GTase inhibitors are promising lead candidates for the development of novel selective capping inhibitors and lead the way to a new class of anti-cancer, antifungal, and antiviral drugs.

What is the biological relevance of the present finding? Numerous studies have demonstrated the potency of MZP to inhibit the cellular IMPDH and to lower the intracellular guanosine nucleotide pool thereby limiting cell growth [28], but none have addressed its impact on the capping apparatus. Monitoring the capping efficiency in living cells is a great challenge as the cellular quality control machinery degrades unsuccessfully capped mRNAs [9], [10], [33]. Since proper capping is crucial for mRNA transcription, export, stability and translation, it is possible to monitor the capping efficiency based on the translation of a reporter protein. In order to evaluate if MZP could impair *in cellulo* capping, we monitored its indirect impact on the translation of the firefly luciferase reporter gene. As cellular protein levels are not only dictated by capping efficiency, we selected a cellular model where all variables unrelated to capping were constant. Cells originating from the same population were trasfected in order to over-express either the active HCE-WT-HA, the GTase-defective HCE-K294A-HA mutant, the GFP control protein or no protein (Control vector). They were submitted to concentrations of 0 µM, 40 µM or 120 µM of mizoribine. All cell lines treated with mizoribine showed a global reduction in reporter protein expression when compared with untreated cells. This expected effect is likely due to partial guanosine pool depletion induced by IMPDH inhibition [28], [34], [35]. Interestingly, the reduction in transcription and translation of the reporter was significantly less severe only in cells over-expression HCE-WT-HA for both mizoribine concentrations. The ability of HCE-WT-HA over-expression to partially rescue the luciferase expression in the presence of mizoribine demonstrates that HCE is one of the mizoribine pharmacological targets. Furthermore, the inability of the GTase defective mutant HCE-K294A-HA to rescue the reporter expression under mizoribine treatment further demonstrates, in agreement with our *in vitro* results, that in a cellular context it is the GTase activity of HCE that is targeted by MZP. Although indirect, this is strong evidence that mizoribine is able to impair capping in a cellular environment. As expected, capping could not be fully inhibited *in cellulo* at mizoribine concentrations of 40–120 µM, which is approximately the *in vitro* IC_50_ of 80 µM. Despite its oral bioavailability (underlying membrane permeability) and its low binding to serum proteins (2.3%), mizoribine was not expected to reach intracellular concentration higher then its IC_50_ [36], [37], [38], [39]. Of notice, the effect of mizoribine on cellular capping was however greater than anticipated. We hypothesize that the competition between HCE and Xrn2 for the nascent mRNA 5′ end could explain the potency of MZP *in cellulo*, as slowing (or inhibiting) the capping activity could be sufficient to shift the balance towards quality control take-over (degradation) and reduce downstream protein expression.

What is the exact contribution of the capping apparatus inhibition to the global mizoribine mechanism of action? This question has yet to be addressed, but the immunosuppressive effect of mizoribine on T-cell is mainly mediated by GTP depletion (which not only blocks T-cells proliferation but also promotes T-cell apoptosis) and might be exacerbated by the reduction of mRNA capping and downstream cap-dependent translation [40], [41]. Our study clearly demonstrates that the therapeutic agent mizoribine monophosphate inhibits the human RNA guanylyltransferase *in vitro* and impairs mRNA capping *in cellulo*.

## Supporting Information

Figure S1 **HCE-C126S inhibition by MZP.** The catalytic cysteine 126 from the conserved RTase motif (I/V)HCxxGxxR(S/T)G was mutated to a serine (HCE-C126S). (A) The RTase activity of the HCE-C126S mutant is nearly abolished as incubation with γ-radiolabeled RNA did not resulted in the release of a significant amount of ^32^Pi (lane 3) when compared to HCE-WT (lane 2). ^32^Pi product was separated from RNA substrate by polyethyleneimine-cellulose TLC plate and quantified by autoradiography. (B) The GTase activity of both the HCE WT and the C126S mutant are inhibited by MZP. A 5′-diphosphate RNA was incubated with [α-^32^P]GTP, 0.1 µM of either HCE-WT or HCE-C126S and increasing concentrations of MZP. Radiolabeled RNA products were analyzed on denaturing polyacrylamide gel and quantified by autoradiography.
(TIF)

Figure S2 **MZP as a potential cap donor substrate for HCE.** A 5′-diphosphate acceptor RNA initiating with a [α-^32^P] guanosine was incubated in absence (lane 1) or in presence of MZP (lane 2) or GTP (lane 3) and HCE. The capping reaction was sequentially submitted to nuclease P1 then alkaline phosphatase prior to analysis by TLC on a polyethyleneimine-cellulose plate. An autoradiogram of the plate is shown. The locations of the origin and GpppG product are indicated by arrows (right).
(TIF)

Figure S3 **HCE does not influence the firefly luciferase activity and stability.** Cellular extract expressing the firefly luciferase protein was incubated alone (white) or supplemented with high concentration (1 µM) of recombinant HCE protein (gray). Alternatively, control cellular extract not expressing the firefly luciferase were incubated in presence of 1 µM of recombinant HCE (black). After the indicated time, the luciferase luminescence was quantified according to the manufacturer instruction (Promega). Luciferase intensity was normalized to the cellular extract expressing the firefly luciferase alone.
(TIF)

Figure S4 **Molecular docking model for the binding of MZP to HCE.** (A) Ribbon representation of HCE in open conformation (homology model) showing the orientation of the docked MZP. (B, C) Two close-up views of HCE active site are also shown, with emphasis on the residues coordinating MZP. (D) Uncluttered representation of HCE active site amino acids showing the orientation of the docked MZP (green) and GMP (blue). (E) Schematic representation of numerous amino acids that are predicted to interact with MZP. The homology model of HCE in open conformation was based on the 1P16-chain-A open conformation GTase using the ModWeb Modeller software. Docking calculations were carried out using the Docking Server software and the Dreiding force field was used for energy minimization of MZP using built-in Chemaxon tools in Docking Server (23). PM6 semi-empirical charges calculated by MOPAC2007 were added to the ligand atoms. Nonpolar hydrogen atoms were merged and rotatable bonds were defined (24). Docking calculations were carried out using the coordinated of the structural model of HCE. Essential hydrogen atoms, Kollman united atom type charges and solvation parameters were added with the aid of AutoDock tools (25). Affinity (grid) maps of 20×20×20 Å grid points and 0.375 Å spacing were generated using the Autogrid program (25). AutoDock parameter set- and distance-dependent dielectric functions were used in the calculation of the van der Waals and the electrostatic terms, respectively. Docking simulations were performed using the Lamarckian genetic algorithm (LGA) and the Solis & Wets local search method (26). Initial position, orientation and torsions of the ligand molecules were set randomly. Each docking experiment was derived from 100 different runs that were set to terminate after a maximum of 2,500,000 energy evaluations. The population size was set to 150. During the search, a translational step of 0.2 Å, and quaternion and torsion steps of 5 Å were applied. The predicted distance are indicated in *Angströms*.
(TIF)

Table S1 **Key interactions between MZP, GMP, and the active site residues of the HCE GTase domain as predicted by the molecular docking model.**
(TIFF)
