# Supplementary figures and images for: The Immunosuppressive Agent Mizoribine Monophosphate Is an Inhibitor of the Human RNA Capping Enzyme
Source: PLoS One. 2013 Jan 17;8(1):e54621. doi: 10.1371/journal.pone.0054621 (PMC3547949; doi:10.1371/journal.pone.0054621)

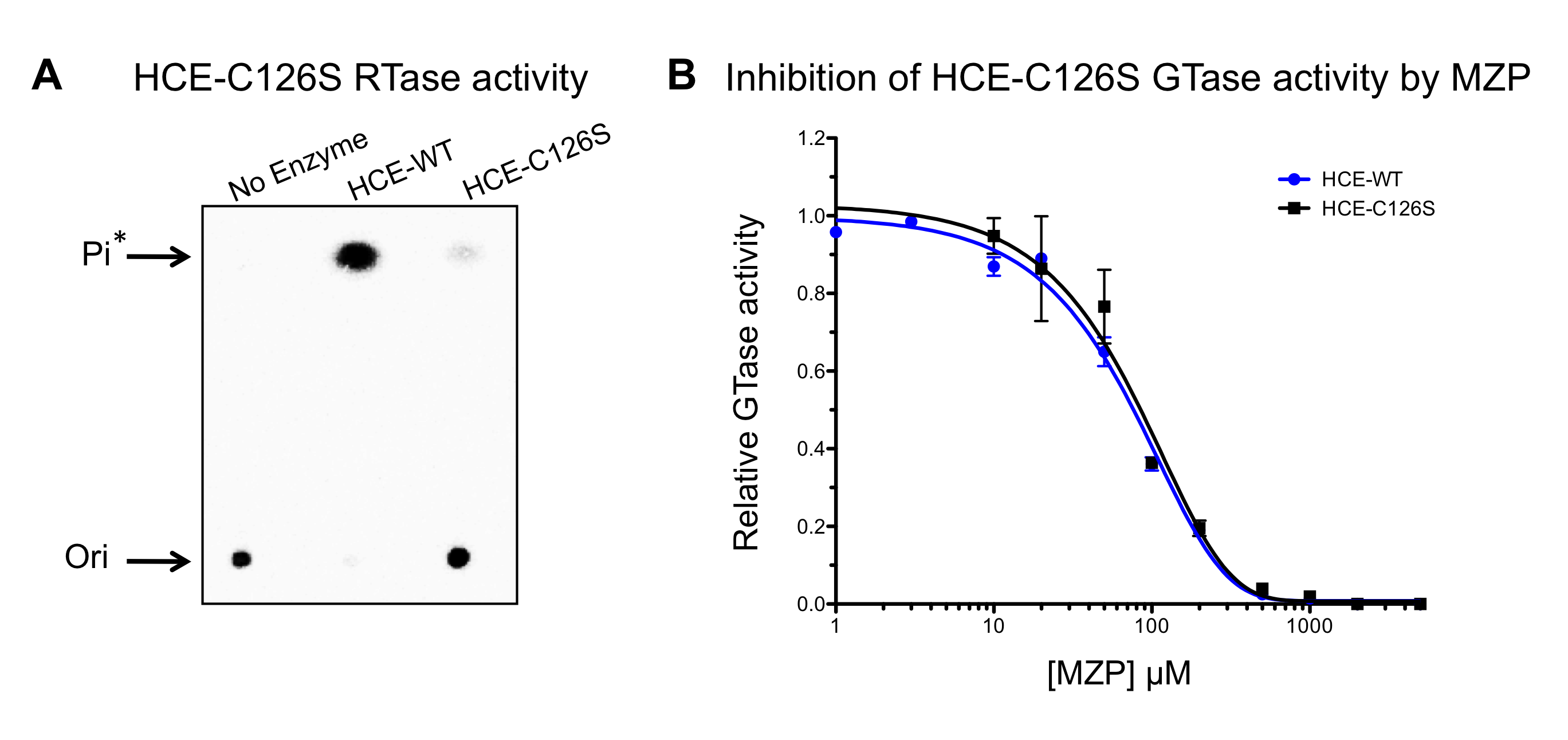

Supplement: Figure S1 — HCE-C126S inhibition by MZP. The catalytic cysteine 126 from the conserved RTase motif (I/V)HCxxGxxR(S/T)G was mutated to a serine (HCE-C126S). (A) The RTase activity of the HCE-C126S mutant is nearly abolished as incubation with γ-radiolabeled RNA did not resulted in the release of a significant amount of 32Pi (lane 3) when compared to HCE-WT (lane 2). 32Pi product was separated from RNA substrate by polyethyleneimine-cellulose TLC plate and quantified by autoradiography. (B) The GTase activity of both the HCE WT and the C126S mutant are inhibited by MZP. A 5′-diphosphate RNA was incubated with [α-32P]GTP, 0.1 µM of either HCE-WT or HCE-C126S and increasing concentrations of MZP. Radiolabeled RNA products were analyzed on denaturing polyacrylamide gel and quantified by autoradiography. (TIF) [file pone.0054621.s001.tif]

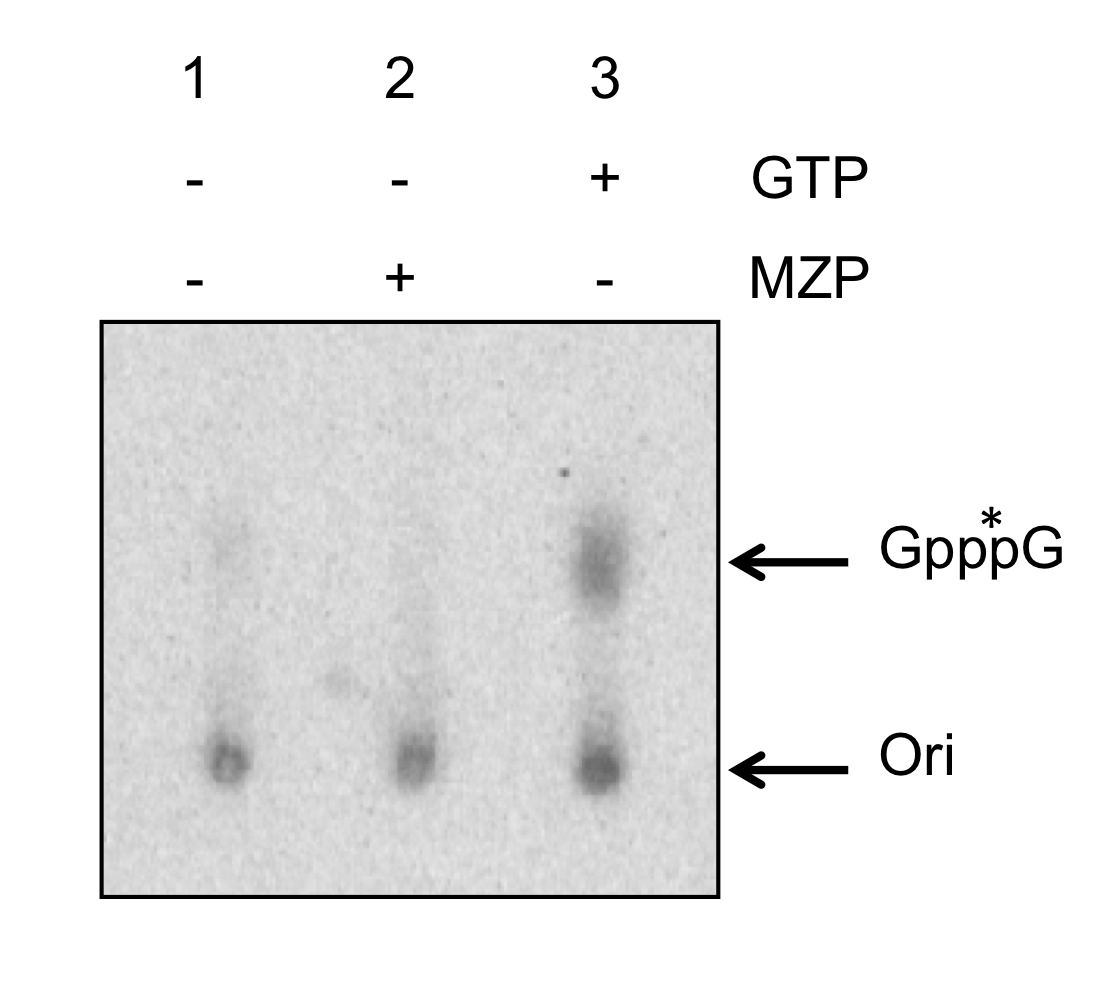

Supplement: Figure S2 — MZP as a potential cap donor substrate for HCE. A 5′-diphosphate acceptor RNA initiating with a [α-32P] guanosine was incubated in absence (lane 1) or in presence of MZP (lane 2) or GTP (lane 3) and HCE. The capping reaction was sequentially submitted to nuclease P1 then alkaline phosphatase prior to analysis by TLC on a polyethyleneimine-cellulose plate. An autoradiogram of the plate is shown. The locations of the origin and GpppG product are indicated by arrows (right). (TIF) [file pone.0054621.s002.tif]

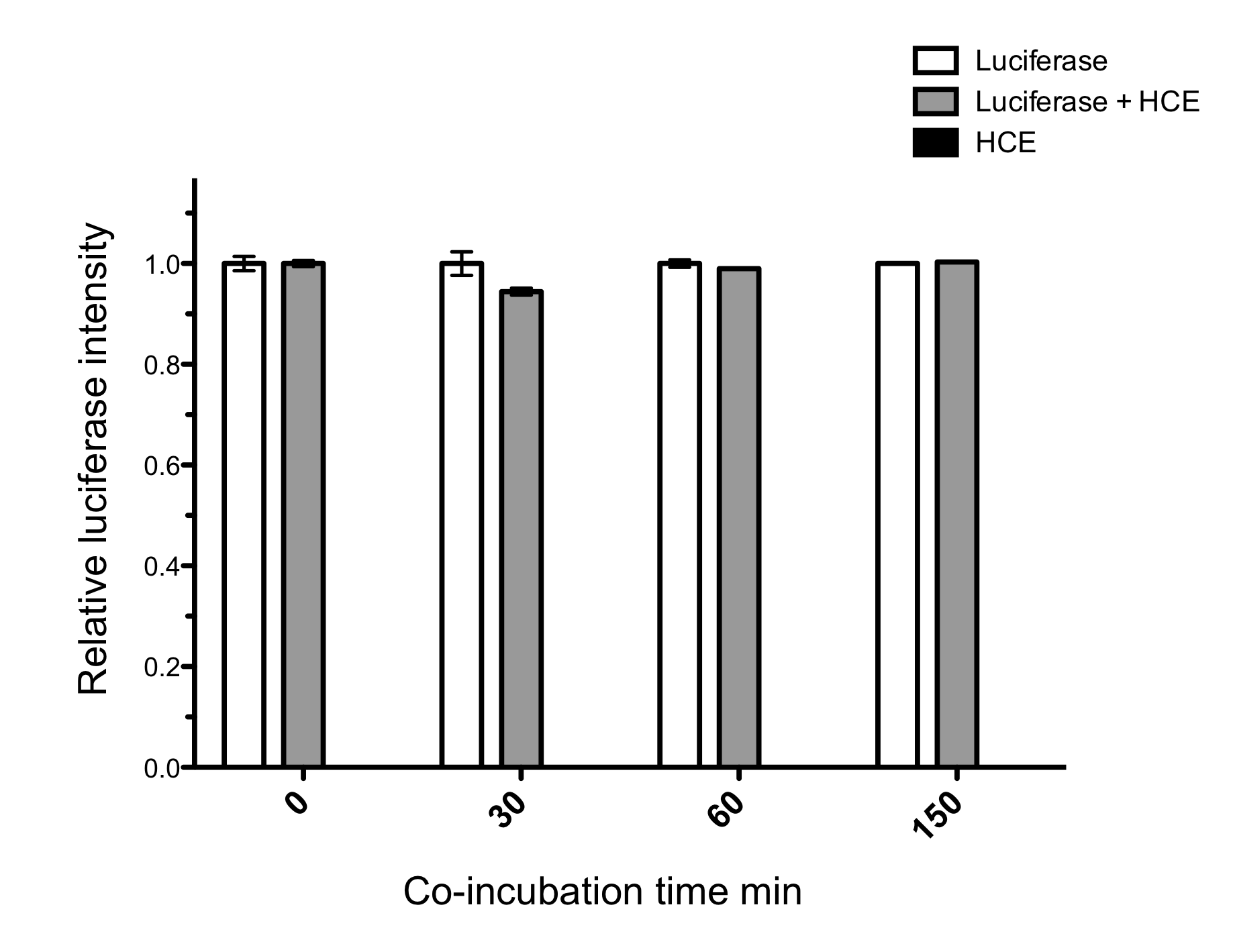

Supplement: Figure S3 — HCE does not influence the firefly luciferase activity and stability. Cellular extract expressing the firefly luciferase protein was incubated alone (white) or supplemented with high concentration (1 µM) of recombinant HCE protein (gray). Alternatively, control cellular extract not expressing the firefly luciferase were incubated in presence of 1 µM of recombinant HCE (black). After the indicated time, the luciferase luminescence was quantified according to the manufacturer instruction (Promega). Luciferase intensity was normalized to the cellular extract expressing the firefly luciferase alone. (TIF) [file pone.0054621.s003.tif]

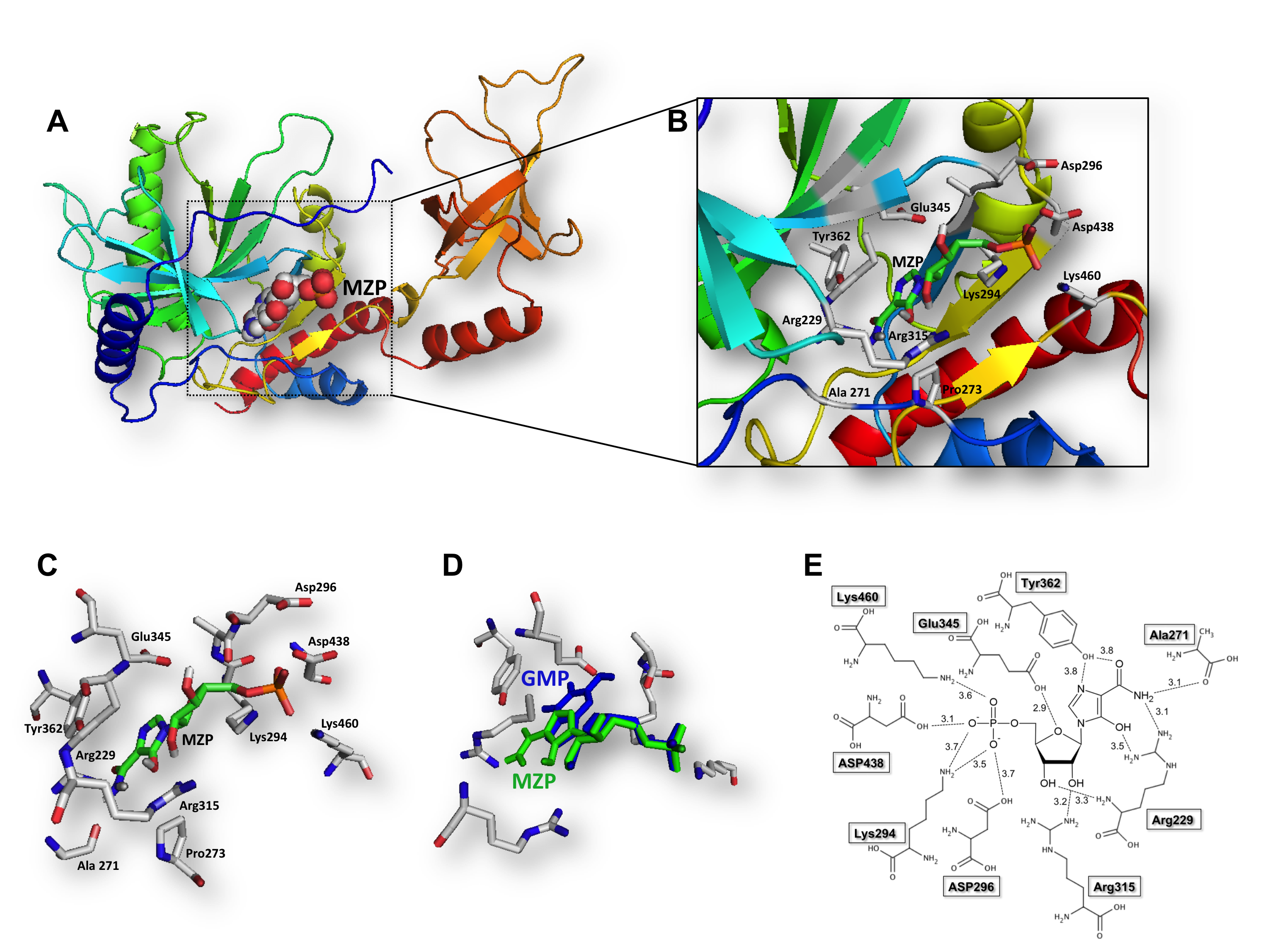

Supplement: Figure S4 — Molecular docking model for the binding of MZP to HCE. (A) Ribbon representation of HCE in open conformation (homology model) showing the orientation of the docked MZP. (B, C) Two close-up views of HCE active site are also shown, with emphasis on the residues coordinating MZP. (D) Uncluttered representation of HCE active site amino acids showing the orientation of the docked MZP (green) and GMP (blue). (E) Schematic representation of numerous amino acids that are predicted to interact with MZP. The homology model of HCE in open conformation was based on the 1P16-chain-A open conformation GTase using the ModWeb Modeller software. Docking calculations were carried out using the Docking Server software and the Dreiding force field was used for energy minimization of MZP using built-in Chemaxon tools in Docking Server (23). PM6 semi-empirical charges calculated by MOPAC2007 were added to the ligand atoms. Nonpolar hydrogen atoms were merged and rotatable bonds were defined (24). Docking calculations were carried out using the coordinated of the structural model of HCE. Essential hydrogen atoms, Kollman united atom type charges and solvation parameters were added with the aid of AutoDock tools (25). Affinity (grid) maps of 20×20×20 Å grid points and 0.375 Å spacing were generated using the Autogrid program (25). AutoDock parameter set- and distance-dependent dielectric functions were used in the calculation of the van der Waals and the electrostatic terms, respectively. Docking simulations were performed using the Lamarckian genetic algorithm (LGA) and the Solis & Wets local search method (26). Initial position, orientation and torsions of the ligand molecules were set randomly. Each docking experiment was derived from 100 different runs that were set to terminate after a maximum of 2,500,000 energy evaluations. The population size was set to 150. During the search, a translational step of 0.2 Å, and quaternion and torsion steps of 5 Å were applied. The predicted distanc [file pone.0054621.s004.tif]
